# Humble Leadership, Psychological Safety, Knowledge Sharing, and Follower Creativity: A Cross-Level Investigation

**DOI:** 10.3389/fpsyg.2018.01727

**Published:** 2018-09-19

**Authors:** Yanfei Wang, Jieqiong Liu, Yu Zhu

**Affiliations:** ^1^School of Business Administration, South China University of Technology, Guangzhou, China; ^2^School of Management, Jinan University, Guangzhou, China

**Keywords:** humble leadership, psychological safety, social information processing, knowledge sharing, follower creativity

## Abstract

The leadership literature has provided growing evidence regarding the power of leadership in facilitating follower creativity. Despite these advances, a noticeable omission in this body of research is humble leadership. The study extends previous research by developing a cross-level moderated mediation model and examining the roles that psychological safety and knowledge sharing may play in the relationship between humble leadership and follower creativity. Using a time-lagged data of 328 team members nested within 106 teams, the results show that: (a) psychological safety mediates the relationship between humble leadership and follower creativity; (b) knowledge sharing moderates the relationship between psychological safety and follower creativity; and (c) the indirect influence of humble leadership on follower creativity through psychological safety is stronger when knowledge sharing is high.

## Introduction

Creativity, defined as the generation of new ideas, has become increasingly critical for organizational performance, survival and success (Anderson et al., 2014). Reflecting the importance of creativity, the past decades have witnessed an upsurge of research interest in identifying factors that contribute to creativity. Most of these studies have stressed that certain leadership styles, such as transformational, empowering, servant, shared, and authentic leaderships, can positively influence follower creativity. Yet despite these advances, a noticeable omission in this body of research is humble leadership (Oc et al., 2015; Wang et al., 2016; Liu et al., 2017). There has been an increasing recognition that leader humility is critical to organizational effectiveness, but only more recently have leadership researchers begun to empirically examine the influence of humble leadership on follower attitudes and behaviors, such as job satisfaction (Owens et al., 2013; Ou et al., 2017), psychological empowerment (Jeung and Yoon, 2016), and job engagement (Owens et al., 2013). To extend this line of research, this study seeks to understand how and when humble leadership influences follower creativity.

Research on social information processing theory provides a theoretical basis for understanding how humble leadership influences follower creativity. Social information processing theory suggests that individuals rely on information cues to understand the work environment and regulate their behaviors (Salancik and Pfeffer, 1978). Leaders serve as a key information source, given their higher status and direct involvement and interactions with followers (Chiu et al., 2016). Therefore, the behavioral modeling of a humble leader in dyadic interactions may shape a psychologically safe environment, where may encourage their creative behaviors. Psychological safety describes the shared perception of the consequences of taking interpersonal risks in their work environment (Edmondson, 1999), and the perception of safe climate is regarded as an important precondition necessary for follower creativity (Carmeli et al., 2010). Therefore, drawing on social information processing theory, we argue that psychological safety may work as a mediator in the relationship between humble leadership and follower creativity.

There are conceptual and empirical reasons to expect that psychological safety has positive influence on follower creativity. However, research on the relationship between psychological safety and creativity has yielded mixed findings (Liu et al., 2016). Some studies suggest that psychological safety does not necessarily lead to creativity (e.g., Li et al., 2015; Liu et al., 2016). Therefore, the relationship described above is apparently more complex than we might expect (Liu et al., 2016). The mixed empirical findings imply the existence of boundary conditions that enable or hinder the positive influence of psychological safety on creativity. A psychologically safe environment provides a basis of interpersonal trust for followers’ engagement in risky creative activities. However, if they do not have enough cognitive capacities to invest in creative activities, creative ideas will be less likely to occur (Yi et al., 2017). Knowledge sharing provides the necessary means for followers to acquire cognitive resources (e.g., ideas, information, and knowledge), thereby expanding cognitive capacities and fostering follower creativity (Carmeli et al., 2013). Therefore, we identify knowledge sharing as one potentially important moderating factor.

Overall, our research aims to develop a cross-level moderated mediation model that explicates how and when humble leadership influences follower creativity. To this end, we incorporate psychological safety and knowledge sharing into a single model, and empirically examine the model using three-wave data.

## Theory and Hypotheses

### Humble Leadership, Psychological Safety, and Follower Creativity

Due to the increasing dynamic and turbulent environment, it is difficult for today’s leaders to figure it all out at the top (Owens and Hekman, 2012). Traditional top-down approach has not been keeping up with the times (Wang et al., 2016). Researchers have suggested that leaders should give up the concept of the “great man,” be open about their limitations in knowledge and experience, and pay more attention to the influence of followers on leader effectiveness (Weick, 2001; Uhl-Bien, 2006). However, despite calls for greater humility in leadership, there are still large gaps in our understanding of how humble leadership may operate in organizations (Owens and Hekman, 2012). Addressing this research gap, this study aims to explore how humble leadership influences follower creativity.

According to social information processing theory, individuals use environmental information cues to construct and interpret events in the workplace and decide how to behave (Salancik and Pfeffer, 1978). Followers tend to gather useful information from their leaders’ statements and behaviors to shape the perception of the work environment, and to act based on the situational desirability of certain behaviors (Lu et al., 2018), because of their leaders’ higher status and direct involvement and interactions with followers (Chiu et al., 2016). As such, when humble leaders admit their limitations and mistakes, appreciate followers’ strengths and contributions, and show teachability (Owens and Hekman, 2012), followers may feel psychologically safe to voice and express new ideas. The perception of safe climate, in turn, may encourage follower creativity. Therefore, drawing on social information processing theory, we propose that psychological safety may be one potential mediator in the relationship between humble leadership and follower creativity.

Specifically, humble leaders not only publicly admit their limitations and mistakes, but also consider mistakes as a normal and even a beneficial part of learning (Owens and Hekman, 2012). Such behaviors send important information that followers can feel psychologically safe to take interpersonal risks and express themselves to realize their potential and grow. This is especially the case when followers under humble leaders have high-quality leader-follower relationships (Owens et al., 2013). The literature indicates that the leader-follower exchange reduces the level of perceived risks and contributes to a strong safety climate (Nielsen et al., 2013). In addition, humble leaders are open to new ideas and suggestions and actively seek for feedback (Owens et al., 2013). This signals to followers that it is safe and even expected to speak up and express new ideas. When humble leaders appreciate followers’ strengths and contributions, they also create a psychologically safe environment because voice is valued and supported, and followers feel able to show and employ one’s self without fear of negative consequences (Nembhard and Edmondson, 2006). Therefore, we expect that humble leadership plays an important role in shaping psychological safety.

Creativity inherently involves high level of challenges, uncertainties and risk, because new ideas are not guaranteed to deliver the desired outcome. In addition, the new ideas generated by followers may not be necessarily encouraged or accepted by their leaders (Zhang and Zhou, 2014). Therefore, a work environment that it is safe to take interpersonal risks and express new ideas is critical for follower creativity, because the environment can motivate and increase one’s willing to show creativity (Carmeli et al., 2010). Specifically, in a psychologically safe environment, they are more likely to take risks and express new ideas because the perception of safe climate allows them to overcome the anxiety and fear of failure (Frazier et al., 2016). In contrast, in a psychologically unsafe environment, they are more likely to develop defensive orientation and are less likely to show creativity at work (West and Richter, 2008). In consistent with our argument, psychological safety has been found to positively influence follower creativity (e.g., Jiang and Gu, 2016).

In sum, the preceding discussion suggests that humble leadership contributes to psychological safety, which in turn encourages follower creativity. We thus expect psychological safety to mediate the influence of humble leadership on follower creativity. In support for the proposal, prior research has indicated that psychological safety plays a mediating role in the relationship between leadership and follower creativity (e.g., Carmeli et al., 2010). Therefore, we hypothesize the following:

**Hypothesis 1:** psychological safety mediates the relationship between humble leadership and follower creativity.

### The Moderating Role of Knowledge Sharing

As noted earlier, creative idea generation is a process of knowledge creation that requires recombining internal and external knowledge into new forms (Radaelli et al., 2014). Internal knowledge is tacit knowledge and expertise that the individual has already possessed, whereas external knowledge is explicit and from others (Carmeli et al., 2013). Therefore, followers never generate new ideas in isolation from other team members, but need to exchange experiences, opinions and information with them (Radaelli et al., 2014). The process where followers mutually exchange their knowledge and information is defined as knowledge sharing (Bai et al., 2016). Considering that the exchange of knowledge is a valuable source of creative ideas (Carmeli and Paulus, 2015), we argue that knowledge sharing may moderate the positive influence of psychological safety on follower creativity. In a psychologically safe environment, the presence of knowledge sharing provides the fundamental means to acquire a wide range of information, knowledge and ideas, which are necessary for follower creativity (Mittal and Dhar, 2015; Bai et al., 2016). In addition, the process of knowledge sharing, beyond the knowledge itself, may also help the generation of creative ideas by facilitating problem identification and enhancing followers’ cognitive abilities (Carmeli et al., 2013; Cheung et al., 2016). More specifically, knowledge sharing allows followers to discuss various problems, thus deepens their understanding of those problems, and helps them identify opportunities for improvement (Cheung et al., 2016). Knowledge sharing also enables followers to fully use internal and external knowledge, thereby enhancing their capacities to develop creative ideas (Carmeli et al., 2013). In contrast, when followers minimally engage in the process of exchange, they may not acquire useful knowledge and information from others. This may be not conductive to followers’ creative engagement, such as processing information and integrating different perspectives, even though they are willing to take risks and express new ideas in a psychologically safe environment. Without additional information, information search and encoding, new ideas will resemble old ideas, thereby leading to less creativity (Carmeli et al., 2013). We thus expect that knowledge sharing and psychological safety should have a joint influence on follower creativity. In other words, follower creativity may be at its highest level when the two conditions are present. Therefore, we propose the following hypothesis:

**Hypothesis 2:** knowledge sharing moderates the relationship between psychological safety and follower creativity, such that the relationship is stronger when knowledge sharing is high rather than low.

The prior arguments represent an integrated framework in which psychological safety mediates the relationship between humble leadership and follower creativity and the influence of psychological safety on follower creativity depends on knowledge sharing. Therefore, we propose the following hypothesis:

**Hypothesis 3:** knowledge sharing moderates the mediating influence of psychological safety on the relationship between humble leadership and follower creativity, such that the mediating role of psychological safety will be stronger when knowledge sharing is high rather than low.

**FIGURE 1:**
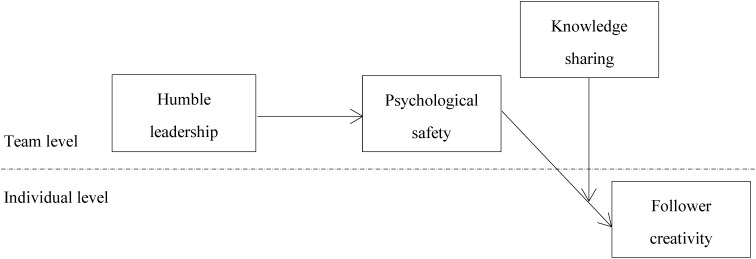
The conceptual model.

Based on the above, we develop a moderated mediation model. **Figure 1** illustrates the theoretical model.

## Materials and Methods

### Participants and Procedure

We conducted our study in Guangdong province, China. Data were collected from employees and their immediate leaders in fifty software firms. With the help of their senior managers, we identified several departments that required substantial creativity as our targeted research sample, such as software research, new product development and quality control. Following Tu and Lu (2013), employees shared the membership of a work group when they reported to the same leader. Therefore, we considered employees and managers in those departments as team members and team leaders, respectively. The survey questionnaires were coded before being distributed so that employees could be matched with their immediate leaders. This was a three-phrase survey with a three-month interval. Specifically, employees were asked to rate their leader’s humble leadership at Time 1, and psychological safety and knowledge sharing at Time 2. On separate questionnaires, the matched leaders rated employee creativity at Time 3. The final sample consisted of 106 team leaders (return rate: 52.2%) and 328 team members (return rate: 71.8%). Of the 106 leaders, 59.5% were male, 68.6% were between 31 and 50 years old, 72.9% had a bachelor degree or above, and 64.4% were with at least 8 tenures. Of the followers, 47.3% were male, 80.7% were younger than 35 years old, 61.3% had a bachelor degree or above, and 54% were with at least 2 tenures.

### Measures

Because the measurements were originally developed in English, we used a standard translation and back-translation procedure to ensure equivalency of meaning. All focus variables were rated by using 5-point Likert-type scales, namely from 1, disagree strongly to 5, agree strongly.

### Humble Leadership

Humble leadership was measured using Owens et al. (2013) nine-item scale. Example items are “this leader is open to the ideas of others” and “this leader often compliments others on their strengths.” The Cronbach’s alpha for this measure was 0.88.

### Psychological Safety

Psychological safety was measured using Edmondson (1999) seven-item scale. Example items are “organizational members are able to bring up problems and tough issues” and “it is difficult to ask other members of my organization for help (reversed).” The Cronbach’s alpha for this measure was 0.76.

### Knowledge Sharing

Knowledge sharing was measured using Lee (2001)’s seven-item scale. Example items are “organizational members share know-how from work experience with each other” and “organizational members share each other’s success and failure stories.” The Cronbach’s alpha for this measure was 0.78.

### Creativity

Creativity was measured using Tierney et al. (1999)’s nine-item scale. A sample item is “this employee tries out new ideas and approaches to problems.” The measure had coefficient alphas of 0.82.

### Control Variables

Following previous research (e.g., Wang et al., 2016), this study includes several control variables. We controlled for gender, age, and tenure under leader at the individual level and team size (i.e., number of teams) at the team level. Followers’ gender, age and team size were controlled for, because they have been found to be related to individual creativity (e.g., Wang et al., 2016). Tenure under leader was included because the literature has suggested that tenure under leader may influence the relationships between humble leadership and individual outcomes (Owens et al., 2013).

## Analytical Approach

Our data were nested in that employees in the same team share the same leader and were thus not independent from one another. To appropriately model the relationships between team-level variables (Level 2) and individual-level variable (Level 1), we conduct a multilevel analysis. In this study, we use multilevel path analysis (Preacher et al., 2010) with Mplus 7.4 (Muthén and Muthén, 2012) to test the multilevel mediation hypothesis. In addition, according to the recommendations of Wallace et al. (2016), we adapt the simultaneous multilevel regression procedure (Bauer et al., 2006) and apply it within Preacher et al. (2010) approach to examine whether knowledge sharing moderates the indirect relationship between humble leadership and follower creativity through psychological safety. To test the significance of the mediation and moderated mediation influences, we employ a Monte Carlo simulation procedure using the open-source software R (Preacher and Selig, 2012).

Since humble leadership, psychological safety and knowledge sharing are considered as team variables, within-group agreement (RWG) and reliability (ICC1 and ICC2) are tested to determine whether the aggregation is appropriate. First, the mean RWG values for humble leadership, psychological safety and knowledge sharing are 0.93, 0.92, and 0.96, respectively, showing a high level of within-group agreement. Second, ICC1 values of humble leadership, psychological safety and knowledge sharing are 0.55, 0.53, and 0.60, respectively, while ICC2 values of these variables are 0.80, 0.78, and 0.82, respectively. Taken together, these results support the aggregation of humble leadership, psychological safety and knowledge sharing.

### Confirmatory Factor Analysis

Before testing the proposed hypotheses, we use Mplus 7.4 to conduct confirmatory factor analyses (CFAs) to examine the discriminant validity of four latent variables: humble leadership, psychological safety, knowledge sharing, and creativity. The results in **Table 1** show that four-factor model [*χ*^2^(389) = 554.352, RMSEA = 0.04, SRMR = 0.05, CFI = 0.95, TLI = 0.94] fits the data better than the three-factor, two-factor, and one-factor models, which supports the variables’ discriminant validity.

**Table 1:** Alternative model test results for the study variables.

Model	*χ*^2^	*df*	Δχ^2^	RMSEA	SRMR	CFI	TLI
Four-factor (expected model)	554.352	389		0.04	0.05	0.95	0.94
Three-factor (humble leadership and psychological safety merged)	645.655	393	91.303^∗∗∗^	0.04	0.06	0.92	0.91
Two-factor (humble leadership, psychological safety, and knowledge sharing merged)	789.894	397	235.542^∗∗∗^	0.06	0.07	0.88	0.86
1-Factor (all items load on a single factor)	1206.558	411	652.206^∗∗∗^	0.08	0.09	0.76	0.73

*The χ^2^ difference was compared with the value of the four-factor model. ^∗∗∗^p < 0.001.*

### Descriptive Statistics

**Table 2** shows the variables’ means, standard deviations, and correlations. As shown in **Table 2**, follower creativity is positively correlated with follower age (*r = −0.11, p < 0.05*). Humble leadership is positively correlated with psychological safety (*r = 0.27, p < 0.01*).

**Table 2:** Means, SD, and correlations of all variables.

Variables	M	*SD*	1	2	3
*Individual level*					
1 Follower gender	0.52	0.50			
2 Follower age	2.45	1.27	−0.05		
3 Tenure under leader	2.87	1.46	−0.04	0.57^∗∗^	
4 Follower creativity	3.56	0.59	−0.09	−0.11^∗^	−0.07
*Team level*					
1 Team size	3.13	0.38			
2 Humble leadership	4.34	0.52	−0.09		
3 Psychological safety	3.64	0.43	−0.01	0.27^∗∗^	
4 Knowledge sharing	4.09	0.38	−0.12^∗^	0.48^∗∗^	0.47^∗∗^

*^∗∗^p < 0.01; ^∗^p < 0.05.*

### Hypothesis Tests

Following recommendations, before testing the cross-level moderated mediation model, individual-level predictors are group-mean centered. Team-level predictors are grand-mean centered. The group-mean centering approach ensures that there is no conflation of the individual and team-level influences in our path analytic results, while grand-mean centering for team-level predictors can diminish the covariance between intercepts and slopes, thereby reducing the potential multicollinearity (Wang and Rode, 2010).

**FIGURE 2:**
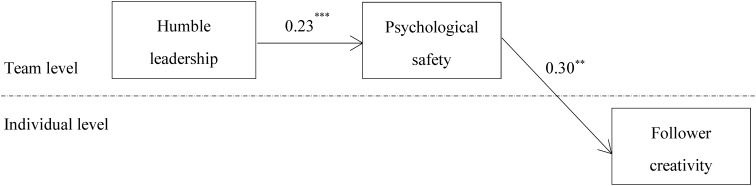
Multilevel mediation model path coefficients. For the sake of parsimony, we did not present the influences of control variables on follower creativity. Interested readers may contact the first author for estimates of these influences. ^∗∗∗^*p* < 0.001; ^∗∗^*p* < 0.01; ^∗^*p* < 0.05.

Psychological safety is hypothesized to mediate the relationship between humble leadership and follower creativity (hypothesis 1). Results (shown in **Figure 2**) from multilevel path analysis support the hypothesized relationship. Specifically, humble leadership is positively related to psychological safety (*b = 0.23, p < 0.001*), and psychological safety is positively related to follower creativity (*b = 0.30, p < 0.01*). To examine the significance of the indirect influence, we use the Monte Carlo simulation procedure with 20,000 replications to construct confidence intervals (CI) for indirect influence. The results show humble leadership has indirect influence on follower creativity via psychological safety (*b = 0.07, 95% CI [0.021 and 0.124]*), showing support for hypothesis 1.

**FIGURE 3:**
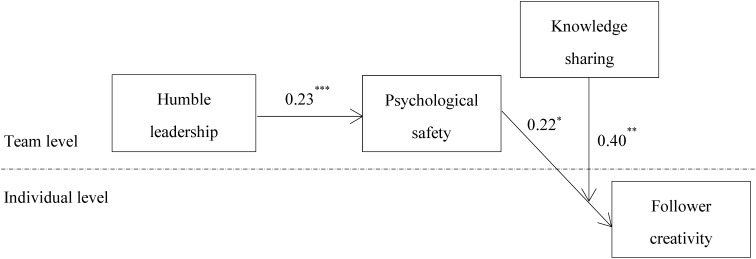
Moderated mediation model path coefficients. For the sake of parsimony, we did not present the influences of control variables on follower creativity. Interested readers may contact the first author for estimates of these influences. ^∗∗∗^*p* < 0.001; ^∗∗^*p* < 0.01; ^∗^*p* < 0.05.

**FIGURE 4:**
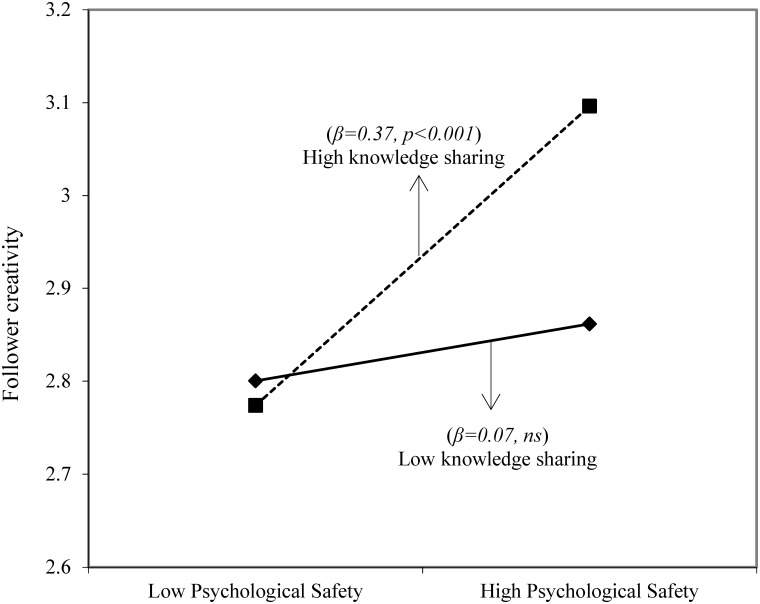
Moderating influence of knowledge sharing on the relationship between psychological safety and follower creativity.

Hypothesis 2 states that knowledge sharing moderates the relationship between psychological safety and follower creativity. As shown in **Figure 3**, all relationships in the proposed moderated mediation model are significant. Furthermore, the multilevel mediation influence does not change substantially after including knowledge sharing in the analysis. Humble leadership is positively related to psychological safety (*b = 0.23, p < 0.001*), while psychological safety is positively related to follower creativity (*b = 0.22, p < 0.05*). In addition, the interaction term (i.e., psychological safety × knowledge sharing) is positive and significant (*b = 0.40, p < 0.01*). To determine the form of the interaction influence, according to the recommendations of Preacher et al. (2006), we plot this interaction influence at one standard deviation above and below the mean of knowledge sharing. The plot in **Figure 4** and the simple slope tests suggest that psychological safety is more positively related to follower creativity when knowledge sharing is high (*b = 0.37, p < 0.001*) than when knowledge sharing is low (*b = 0.07, ns*). Therefore, hypothesis 2 is also supported.

Hypothesis 3 predicts that knowledge sharing moderates the mediating influence of psychological safety on the relationship between humble leadership and follower creativity. The Monte Carlo simulation procedure with 20,000 replications is used to examine the moderated mediation influence. The results show that the indirect influence of humble leadership on follower creativity through psychological safety is significant when knowledge sharing is high (*b = 0.08, SE = 0.03, 95% CI [0.034 and 0.141]*), and it becomes non-significant when knowledge sharing is low (*b = 0.02, SE = 0.03, 95% CI [-0.049 and 0.079]*), supporting hypothesis 3.

## Discussion

The study explores how and when humble leadership influences follower creativity. Applying a three-phrase research design, we find support for our hypotheses. First, based on social information processing theory, we have found the key role of psychological safety in linking humble leadership and follower creativity. This finding is consistent with most of the previous work that shows the mediating influence of psychological safety on the relationship between leadership and psychological safety (e.g., Carmeli et al., 2010). Humble leaders admit their mistakes, are open to new ideas and suggestions, actively seek for feedback, tend to develop high-quality leader-follower relationships, and appreciate followers’ strengths and contributions. According to social information processing theory, the behavioral modeling in the humble leadership process shapes followers’ shared perceptions about their work environment that it is safe and even expected to speak up and express new ideas without fear of negative consequences. Followers in the safe environment are willing to take risks and express new ideas because the perception of safety climate allows them to overcome the anxiety and fear of failure.

Second, we have also found that psychological safety may not help follower creativity when knowledge sharing is missing. Creative idea generation is a process of knowledge creation that requires recombining internal and external knowledge into new forms. Without relevant information and inputs from others, followers are less likely to generate new ideas. The process of knowledge sharing provides the fundamental means to acquire a wide range of information, knowledge and ideas, which helps broaden their cognitive resources and capacities. Therefore, it is very likely that follower creativity will be at its highest level when both psychological safety and knowledge sharing are present. This result is aligned with the earlier argument that psychological safety alone may not lead to team learning, unless conditions that call for learning and communication are present (Kostopoulos and Bozionelos, 2011; Edmondson and Lei, 2014).

Third, knowledge sharing has been found to moderate the mediating role of psychological safety in the relationship between humble leadership and follower creativity. The finding indicates the importance of knowledge sharing as a boundary condition for the effectiveness of humble leadership in fostering follower creativity. More specifically, in such a situation that there is no or little knowledge sharing among team members, followers are less likely to generate new ideas, even though humble leaders create salient social cues to team members that it is safe to speak up and express new ideas without fear of negative consequences. However, followers’ perception of safety climate shaped by humble leaders will encourage their creativity when knowledge sharing is present.

### Theoretical Implications

Our findings make several important contributions to present knowledge. First, our research contributes to humble leadership literature by answering the calls of Jeung and Yoon (2016) and Yuan et al. (2018) to examine the psychological mechanisms between humble leadership and follower creativity. The leadership literature has provided several theoretical perspectives to explain how leadership influences follower creativity, such as intrinsic motivation and social learning. However, these studies have mainly focused on top-down leadership and the bottom-up aspects of leadership have been largely overlooked (Oc et al., 2015; Wang et al., 2016; Liu et al., 2017). Distinct from top-down leadership, humble leadership is a new type of bottom-up leadership style distinctly characterized by admitting personal limitations, publicly praising followers, and maintaining an open mind (Owens and Hekman, 2012). Although early propositions have pointed to the importance of leader humility within organizations, studies toward exploring the influence of humble leadership on followers – and the underlying mechanisms involved – have only recently begun to attract attention (e.g., Jeung and Yoon, 2016; Wang et al., 2016; Liu et al., 2017; Ou et al., 2017, 2018; Rego et al., 2017; Yuan et al., 2018). Furthermore, these studies are primarily limited to the individual-level or team-level analysis. A focus on just one level is likely to provide an incomplete, or even inaccurate, understanding (Edmondson and Lei, 2014), because the cross-level influences may demonstrate “spillover” influences of group-focused leadership *down to* individual-level outcomes (Li et al., 2014, 2015). Therefore, we move beyond previous research by examining the cross-level indirect influence of humble leadership on follower creativity via psychological safety, thereby leading to a more precise specification of outcomes and processes.

Second, our research contributes to the current literature of psychological safety and creativity by exploring the potential boundary conditions for the influence of psychological safety on creativity. Research on the relationship between psychological safety and creativity has yielded mixed findings (Liu et al., 2016), which indicates that although psychological safety has often been identified as a predictor of creativity, it also interacts with other variables to alter predicted relationships (Edmondson and Lei, 2014). However, research on the boundary conditions associated with the influence of psychological safety remains underdeveloped (Edmondson and Lei, 2014). Clarifying the dynamics of the complex relationship between psychological safety and creativity is vital to advance the theorizing about the influence of psychological safety on creativity (Chen et al., 2016). Therefore, in an attempt to expand this line of research, we examine and demonstrate the moderating role of knowledge sharing in the relationship between psychological safety and creativity. The results provide an account of the complex relationship that knowledge sharing moderates the influence of psychological safety on creativity, and only when knowledge sharing is present can psychological safety positively influence creativity.

Third, our research also contributes to the leadership literature by identifying boundary conditions for the indirect influence of humble leadership on follower creativity via psychological safety. Although most research has indicated the mediating role of psychological safety in the relationship between leadership or behaviors and follower creativity (e.g., Carmeli et al., 2010), Li et al. (2015) found no evidence for such a mediation process. Some scholars have built a sequential mediational model to account for the inconsistent findings in the literature (e.g., Liu et al., 2016; Gonçalves and Brandão, 2017). For example, Gonçalves and Brandão (2017) found that psychological safety mediates the relationship between humble leadership and psychological capital, which is then positively related to team creativity. This study extends the recent empirical work by incorporating potential mediator (i.e., psychological safety) and moderator (i.e., knowledge sharing) into a single framework and developing a cross-level moderated mediation model to disentangle the complexity and advance our understanding of humble leadership. More specifically, the finding that the conditional indirect influence of humble leadership on follower creativity, via psychological safety, differs in strength across low and high levels of knowledge sharing broadens existing knowledge on whether and when psychological safety functions as a mediator linking humble leadership and follower creativity.

### Practical Implications

These findings may have several practical implications. First, our findings point to the importance of humble leadership for facilitating follower creativity. Humility is a valuable virtue that may be learned and developed (Rego et al., 2017). Therefore, leadership training and development programs should be provided to help leaders understand the importance of humility and develop their humility. In addition, using humility as a selective criterion for future leaders may also be an effective measure. Second, our findings also suggest that psychological safety works as a mediator linking humble leadership and follower creativity. Therefore, organizations should pay much attention to creating conditions where followers feel safe to take risks and bring up new ideas. The literature indicates that some actions taken by organizations and leaders are beneficial for the development of psychological safety, such as encouraging followers, appreciating their contributions, and building trust and supportive relationships with them. Third, knowledge sharing is found to moderate the influence of psychological safety on follower creativity. The finding indicates that psychological safety could not always warrant the production of creative outcomes. Under some specific conditions (i.e., knowledge sharing is missing), the positive influence of psychological safety on follower creativity may disappear. Only when knowledge sharing is present can psychological safety positively influence follower creativity. Therefore, organizations should be able to receive high level of creativity if their attention is directed toward creating conditions that psychological safety and knowledge sharing are simultaneously present.

### Limitations and Future Directions

In this study, several limitations should be noted. First, humble leaders have a greater tendency to acknowledge strengths. Whether there was response bias in the leaders’ ratings of creativity remains a question. Therefore, although we have excluded the potential influence of common method bias by collecting the data from two different sources, which is the practice of most creativity research (Shin et al., 2012), it is still hard for us to infer the causal relationship. Future research is suggested to employ objective measures of follower creativity to strengthen causal inference. Second, our findings indicate that psychological safety only has partial mediating influence on the relationship between humble leadership and follower creativity. That is, other potential mediating mechanisms underlying this relationship have been excluded from the study. Therefore, future research should explore a broader range of mediating mechanisms through which climate for innovation impacts positively on employee creativity. Third, this study was conducted in the Chinese context with a high collectivist and high-power distance culture, which may raise the question about generalizability of our findings to Western and other cultural contexts. Considering that humility is culturally bound and that the potentially unique importance of humility in collectivistic or high-power distance cultures (Oc et al., 2015), additional research is needed to test if the findings of our study can be generalized across cultures.

## Ethics Statement

An ethics approval was not required as per institutional guidelines and national laws and regulations because no unethical behaviors existed in this study. We just conducted paper-pencil test and were exempt from further ethics board approval since our study did not involve human clinical trials or animal experiments. In the survey process, all participants were informed that participation was voluntary and assured that their responses would be only used for our research and kept confidential strictly. Therefore, only those who were willing to participate were recruited. To ensure confidentiality, the questionnaires completed during their working hours were directly returned to these research assistants in sealed envelopes.

## Author Contributions

YW provided the idea, designed this study, and wrote the manuscript. JL and YZ contributed to research design and data collection and analysis. All authors contributed equally to this paper and reviewed and approved this paper for publication.

## Conflict of Interest Statement

The authors declare that the research was conducted in the absence of any commercial or financial relationships that could be construed as a potential conflict of interest. The reviewer YS and handling Editor declared their shared affiliation.
